# Monitoring Immune Dysfunction in Critically Ill Patients with Carbapenem-Resistant *Acinetobacter baumannii* Sepsis Treated with Regimens Including Cefiderocol: A Pilot Study to Identify Accessible Biomarkers to Stratify Patients’ Prognosis

**DOI:** 10.3390/antibiotics13111001

**Published:** 2024-10-23

**Authors:** Antonella Frattari, Ennio Polilli, Laura Timelli, Francesca Spagnuolo, Paolo Fazii, Giustino Parruti

**Affiliations:** 1 Intensive Care Unit, Pescara General Hospital, 65100 Pescara, Italy; antofrattari@gmail.com (A.F.); francesca.spagnuolo@asl.pe.it (F.S.); 2 Clinical Laboratory Unit, Pescara General Hospital, 65100 Pescara, Italy; en.polilli@gmail.com; 3 Independent Researcher, 00100 Rome, Italy; timelli.laura@gmail.com; 4 Clinical Microbiology Unit, Pescara General Hospital, 65100 Pescara, Italy; paolo.fazii@asl.pe.it; 5 Infectious Diseases Unit, Pescara General Hospital, 65100 Pescara, Italy

**Keywords:** sepsis, Cefiderocol, treatment failure, multidrug-resistant *Acinetobacter baumannii*, immune paralysis

## Abstract

**Background:** Multidrug-resistant *Acinetobacter baumannii* (CRAB) infections are a serious problem in critical care. This study aims to develop an early prognostic score for immune paralysis, using practical and cost-effective parameters, to predict ICU mortality in patients with CRAB infections being treated with Cefiderocol. **Methods:** We carried out an observational pilot study on consecutive patients hospitalized in the ICU with ensuing septic *Acinetobacter baumannii* infections treated with Cefiderocol monotherapy or Cefiderocol including combinations. We investigated the predictive power of lymphocyte counts, lymphocyte subpopulations, serum cholinesterase levels, and reactivation of herpes viruses. **Results:** Overall, 36 of 39 patients entered in our analysis: 20 survivors and 16 deceased. A total of 12 patients developed bacteremia, 19 patients had HAP/VAP, and 5 patients had a soft tissue infection. Univariate analyses of factors associated with unfavorable outcome revealed a significant association for age (OR: 1.5, CI: 1.11–2.02), SAPS II (OR: 1.05, CI: 1.01–1.1), SOFA score (OR: 1.37, CI: 1.06–1.76), lymphocytopenia (OR: 32.5, CI: 3.45–306.4), viral reactivation (OR: 9.75, CI: 1.72–55.4), and cholinesterase drop <1600 U/L (OR: 39.7, CI: 5.8–271.6). At variance, monotherapy or associations with Cefiderocol were not associated. In the final multivariable model, the only independent predictors of death were age (OR: 1.42, CI: 0.98–2.05), lymphocytopenia (OR: 18.2, CI: 0.87–371), and cholinesterase drop to below 1600 U/L (OR: 9.7, CI: 0.77–123.7). **Conclusions:** Age, lymphocytopenia, and serum cholinesterase drops, which were nearly significantly associated with an unfavorable outcome, may help pinpoint patients with acute immune paralysis during sepsis. Knowledge of such an immune state may in turn directly influence patients’ care.

## 1. Introduction

Sepsis, an acute organ dysfunction induced by dysregulated response to infection, is the major cause of death in the Intensive Care Units (ICUs). Primary site of infection, type of pathogen involved, severity of acute organ dysfunction, previous health status, and possible delay in the initial antimicrobial therapy are all factors that determine the clinical appearance, severity, and prognosis of sepsis [1,2,3,4]. Despite improvements in critical care, including early and appropriate treatment initiation for underlying infection(s) and support of failing organs, mortality is still high due to dysregulated host responses [5,6,7]. Multiple drug-resistant organism (MDRO) colonization and ensuing infections by carbapenem-resistant *Acinetobacter baumannii* (CRAB) may occur in patients with multiple comorbidities, exposure to invasive procedures, prolonged ICU stay, and antibiotic therapy [8,9]. CRAB infections are difficult to treat, with treatment options being limited due to intrinsic and acquired resistance to several antimicrobial classes, so all-cause mortality rates range between 18% and 57% in such patients [10,11,12]. Indeed, CRAB represents a threat to the most vulnerable patients, leading to significantly higher mortality rates than in those infected with susceptible strains, in spite of similar comorbidities and risk factors [13,14,15]. Cefiderocol, a novel siderophore cephalosporin, has been approved to treat serious infections caused by carbapenem-resistant Gram-negative bacteria and represents an interesting new therapeutic option for the treatment of serious infections caused by CRAB. While the phase 3 randomized clinical trial CREDIBLE-CR, comparing Cefiderocol with the best available therapy, showed higher mortality rates in patients treated with Cefiderocol than in controls [16], subsequent real-world reports from case series and observational studies on the use of Cefiderocol in the ICU for serious CRAB infections showed promising results, both in terms of efficacy and safety [17,18]. The advantage in terms of survival was more evident in patients with bloodstream infections than in those with VAP [19,20], probably due to the suboptimal penetration of Cefiderocol in the ELF at current dosages [21]. Treatment failure, however, may occur both in patients treated with Cefiderocol monotherapy and in those treated with various antibiotic combinations in relation to other factors, such as acute suppression of the host’s immune system [22]. Even before the SARS-CoV-2 pandemics, we were interested in gathering information on the possible role of acute immune paralysis in ICU patients [23]. In the present pilot study, we gathered information on easily measurable bedside parameters that may contribute to defining a prognostic measure of immune paralysis to predict ICU mortality among patients with sepsis or severe CRAB infections treated with Cefiderocol monotherapy or antibiotic combinations including Cefiderocol.

## 2. Results

From September 2022 until January 2024, 39 out of 696 patients (5.4%) assisted at our Intensive Care Unit for at least 48 h developed serious *Acinetobacter baumannii* infections and sepsis and were treated with antibiotic regimens that included Cefiderocol. Three patients were excluded from the final sample because of previously diagnosed multiple myeloma (1) and a supervening SARS-CoV-2 infection (2). The final sample, therefore, included 36 patients, all Caucasians: 20 survivors and 16 deceased (44.4%) at 30 days after the ensuing infection. Among these, 12 patients developed *Acinetobacter baumannii* bacteremia, 19 patients had HAP/VAP, and 5 patients presented an *Acinetobacter baumannii* soft tissue infection during their ICU stay (Table 1). For all patients, but in three in the survivor group, sepsis was diagnosed in accordance with Sepsis 3 criteria. Age was significantly higher (73 y, r. 64.5–78 vs. 52 y, r. 40.5–68.5, *p* = 0.003) among non-survivors, as was SOFA score (8, r. 6–11.5 vs. 5.5, r. 4–7.5, *p* = 0.018) and SAPS II score (51, r. 35–64 vs. 38.5, r. 28–46.5, *p* = 0.048). Males were more frequent both among survivors and deceased (*p* = 0.67). There was no difference in survival for patients treated with monotherapy versus combination therapy that included Cefiderocol (10 monotherapies and 10 combinations among non-survivors, 9 monotherapies and 7 combinations among survivors, *p* = 0.709). At variance, among patients with persistently normal lymphocyte counts or reverted lymphocytopenia, there were 19 survivors vs. 3 deaths, whereas among patients with profound (<400/cells/µL) and persistent lymphocytopenia, there were 0 survivors and 13 deaths, *p* < 0.001. Among patients with a progressive and persistent drop in serum cholinesterase values up to below 1600 U/L, there were 3 survivors and 14 deaths, *p* < 0.001; similarly, among patients with reactivation of CMV and/or HSV1 viruses, there were 2 survivors and 13 deaths, *p* = 0.005. Neither clinical nor laboratory signs of potential toxicity or idiosyncrasy to Cefiderocol were evidenced in any of the included patients.

Univariate analyses of factors associated with unfavorable outcome at 30 days revealed a significant association for age (for 5 y increase, OR: 1.5, CI: 1.11–2.02, *p* = 0.008), SAPS II score (for 1 point increase, OR: 1.05, CI: 1.01–1.1, *p* = 0.042), SOFA score (for 1 point increase, OR: 1.37, CI: 1.06–1.76, *p* = 0.017), lymphocytopenia (present or absent, OR: 32.5, CI: 3.45–306.4, *p* = 0.002), viral reactivation (OR: 9.75, CI: 1.72–55.4, *p* = 0.01), and cholinesterase drop up to <1600 U/L (OR: 39.7, CI: 5.8–271.6, *p* < 0.001; Table 2). In the final multivariable model, the only independent predictors of death were age (for 5 y increase, OR: 1.42, CI: 0.98–2.05, *p* = 0.063), lymphocytopenia (OR: 18.2, CI: 0.87–371, *p* = 0.062), and a cholinesterase drop up to <1600 U/L (OR: 9.7, CI: 0.77–123.7, *p* = 0.078). The AUC of the model was 0.95 (95% CI: 0.87–1.0), and the p-value of the Hosmer and Lemeshow test was 0.94, implying that the model’s estimates fit the data well (Table 2).

## 3. Discussion

The host response to infection is a dynamic and complex process. Sepsis-induced immunosuppression has been acknowledged and linked to worse outcomes and increased healthcare costs [24,25]. Moreover, a marked suppression of the immune response has also been described in patients hospitalized in the ICU for severe trauma or other critical conditions [26,27,28]. Although a clear definition for such injury- and sepsis-induced immunosuppression is yet lacking, it has been hypothesized that immune monitoring could identify patients who might benefit most from novel, adjunctive immune-stimulating therapies [29,30,31,32]. Immunosuppression is associated with frequent viral reactivations (e.g., CMV, EBV, HSV), an increased susceptibility to secondary infections by both common pathogens and opportunistic microorganisms, innocuous for hosts. In the critical care literature, the term “immunocompromised” generally refers to patients with at least one cause of immune compromise upon ICU admission, including primary inherited and acquired immunodeficiencies due to cancer, hematologic malignancies and neutropenia, solid organ transplantation, long-term treatments with steroids and/or other immunosuppressive drugs, or untreated HIV infection [31,33,34]. Conversely, patients without clinical or biological evidence of immunosuppression are often, by default, considered immune-competent, a questionable assumption among those with ensuing sepsis and dysregulated host responses [31,34]. Indeed, multiple conditions altering immuno-inflammatory responses may well determine a covert immunosuppression [31]. Critically ill patients in the first waves of COVID-19 were generally deemed ‘immunocompetent’, in spite of harboring multiple comorbidities such as obesity, diabetes, and cardiovascular disease [31,35]. During the COVID-19 pandemics, MDR organisms, particularly CRAB, were reported as causative agents of secondary infections, especially in severe and critical cases [19,36]. CRAB acquisition in the ICU increased in parallel with the length of stay and accounted for higher mortality [19,36]. As of yet, however, no validated diagnostic test is available to assess the actual state of immunosuppression in critically ill patients otherwise deemed as immune-competent upon admission to the ICU [31]. In spite of the current dearth of data, some markers of acute immune dysfunction in critically ill patients are supported by sufficient evidence that can be tentatively used to define a better stratification of the risk of immune paralysis in individual patients [37]. We investigated potentially relevant immunological parameters to define acute immune suppression, such as daily lymphocyte counts [36,38,39,40], assays of lymphocyte subpopulations upon ICU admission [41,42], and serum cholinesterase levels [43,44,45], for all of which supportive literature evidence is present. We also repeatedly monitored the reactivation of herpes viruses during ICU stay as a possible signal of acute immune imbalance of adaptive immunity [25,35,46,47]. Our study revealed that a remarkable proportion of non-survivors presented persistent herpes virus reactivation during their ICU stay, significantly associated with an unfavorable outcome at univariate analyses. The clinical significance of viral reactivation is unclear, as direct organ involvement is uncommonly encountered [46,47]. Viral reactivation, however, may have immunomodulatory consequences, increasing the risk of ICU-acquired bacterial and fungal infections [48]. Herpes virus reactivation failed to independently predict an unfavorable outcome in multivariate models. In our pilot study, at variance, two easily assessable parameters were strictly linked to death in the multivariate models. The first was an absolute and persistent drop in lymphocyte counts below 500 at serial, daily assays. This biomarker of immune function is a direct measure of the drop in adaptive immunity due to apoptotic lymphocyte death as a consequence of persistent immune activation [31]. Notably, early and persistent lymphopenia may be a robust signal of immunosuppression in critically ill patients, associated with poor prognosis [36,38,39,40,49]. In our previous work on a large series of COVID patients assisted at our ICU, multivariate analyses revealed a significantly increased risk of mortality for lymphocytopenia (OR: 2.32, 95% CI: 1.49–3.64, *p* = 0.0002), ensuing bacteremia (OR: 2.05, 95% CI: 1.31–3.22, *p* = 0.0014), and reactivation of herpes viruses (OR: 2.29, 95% CI: 1.29–4.19, *p* = 0.0057) [36]. Several other studies in recent years confirmed that profound and persistent lymphocytopenia may well be associated with an unfavorable outcome in patients with sepsis, even when considered as a single biomarker [38,39,40]. In addition, persistent cholinesterase drops during sepsis are another easily assessable and interesting biomarker of dysregulated inflammation caused by sepsis [43,44,45]. Serum cholinesterase concentrations are linked to immune modulation of inflammatory responses, and reductions in serum cholinesterase levels, in the absence of other signs of hepatic dysfunction, have been reported in many studies on septic patients [43,44,45]. Indeed, serum cholinesterase levels are modulated during sepsis to increase the power of cholinergic quenching of inflammation at peripheral sites in the immune system [43,44,45]. So, without surprise, in our small sample of patients with CRAB septic infections, we found that a profound and persistent drop in serum cholinesterase levels could predict unfavorable outcomes in association with persistent lymphocytopenia in multivariable models. Interestingly, the area under the ROC curve revealed that such combined prediction, in association with age, was strong enough to lay the basis for a practical predictive score in the absence of adjunctive parameters in larger series. Indeed, in our present series, we observed no difference in survival between patients treated with Cefiderocol monotherapy and those treated with associative regimens that included Cefiderocol. One of the major drivers of mortality in septic patients infected with *A. baumannii* is inappropriate initial antibiotic therapy, mainly due to the high resistance of CRAB isolates, for which an established consensus on the optimal treatment is yet lacking [10,13]. Our pilot data suggest that the assessment of other factors mirroring the state of actual immune imbalance in septic patients may shed light on such a complex conundrum.

Our work has several relevant limitations. First, it was a small, single-center study, carried out on a small number of consecutive patients with severe CRAB infections and sepsis, which limits the relevance of any possible conclusion to be drawn. Second, we were unable to assess the actual sensitivity of the isolated strains of CRAB to Cefiderocol, which may have influenced our preliminary results. Third, due to the paucity of patients in our sample, associations of the potentially independent predictors of an unfavorable outcome were all near significant in the final multivariable model. Notwithstanding such limitations, our pilot data may shed light on the need to investigate additional factors, such as easily assessable biomarkers of the patients’ acute immune dysregulation, when evaluating the efficacy of any antibiotic treatment for severe CRAB infections, a working hypothesis that deserves to be evaluated on a larger and multicentric series of critically ill patients. Several other immune biomarkers recently emerged as possible tools to quantify the risk of immune paralysis, such as monocyte expression of human leukocyte antigen class II histocompatibility DR molecules (HLA-DR) [50] and other immune functional tests, including assays of immunoglobulin levels [30,51]. However, most of these biomarkers may have limited widespread availability for ICU patients [37,52]. Furthermore, many interventions in the ICU, including invasive procedures (intravascular catheters, endotracheal intubation, and mechanical ventilation) and drugs such as corticosteroids, sedatives, and catecholamines, have potent immunomodulatory properties [53,54]. Similarly, blood products, including concentrates of red blood cells, platelets, and fresh–frozen plasma, may alter the host’s immune response with complex mechanisms [55,56]. Finally, some antibiotics could alter immune responses through mitochondrial toxicity [55,56]. Administration of Cefiderocol, at variance, might protect against cytokine- and endotoxin-mediated endothelial damage, as demonstrated by in vitro models, although its efficacy in vivo may be reduced in critically ill patients, whereby plasma proteins and acute phase reactants may quench the expression of membrane siderophores for iron uptake in *Acinetobacter baumannii* circulating strains [51,57]. The relevance of all these findings in critically ill patients, however, is yet unclear, which leaves the opportunity for our findings to be tested in a larger series. In particular, it will be interesting to test the working hypothesis that adjunctive immune therapies, such as early administration of immunoglobulin concentrates in patients at high risk of ongoing immune paralysis, may be able to ameliorate such an imbalance and ease bacterial clearance, at least in those with low immunoglobulin levels at ICU entrance [58].

## 4. Conclusions

Our experience was based on a small series of consecutive, critically ill patients with severe infection/sepsis caused by multidrug-resistant *Acinetobacter baumannii* during their stay in the ICU, well characterized by predictors of acute immune imbalance alongside demographic, clinical, biochemical, and microbiological parameters, measured before and after ensuing infection and sepsis due to CRAB. Mortality rates at 30 days were high (44.5%), in spite of prompt therapy with Cefiderocol or Cefiderocol-containing antibiotic combinations. Among potential predictors of mortality, age, lymphocytopenia, and serum cholinesterase drops were independently and near significantly associated with an unfavorable outcome, indicating that such easily measurable parameters should be taken into account when caring for patients in the ICU to recognize the need for possibly adjunctive immune therapies. A promising and simple approach to immune monitoring, catching clinically relevant immune alterations during ICU stay, may directly influence patients’ care, either through inducing enhanced clinical surveillance, early start of antimicrobials in the suspicion of infection, removal of potentially superfluous invasive devices, or immune stimulation strategies. Further investigations on larger and multicentric prospective series are warranted.

## 5. Patients and Methods

We carried out a single-center, observational pilot study on consecutive patients hospitalized in the Intensive Care Unit of Pescara General Hospital with ensuing *Acinetobacter baumannii* infections between September 2022 and January 2024. The study was carried out in accordance with the Declaration of Helsinki (amended version). The local Health Administrative Board of Pescara General Hospital (PGH) authorized a daily collection of anonymized data for the monitoring of local outcomes and research purposes in daily ICU practice so that no further authorization was required ahead of retrospective data collection. For each included patient, we recorded sex, age, ICU admission diagnosis, underlying diseases, infection site, Charlson’s Comorbidity Index, SOFA score, SAPS II score, daily counts of peripheral blood lymphocytes, available determinations of procalcitonin, C-reactive protein, serum cholinesterase, monocyte distribution width (MDW), as well as blood, rectal, pulmonary, and skin microbiological isolates. Assays of peripheral blood lymphocyte subsets were performed at the ICU entrance within 2 days, and both absolute CD4 T-cell counts and the ratio of the percentages of CD8 T-cells and CD19 B-cells were considered, in accordance with a previous report [41,42]. Herpes virus (HSV1, HSV2, VZV, CMV, EBV, HHV6) reactivation was monitored weekly on peripheral blood from ICU admission until discharge. Ensuing sepsis or septic shock due to *Acinetobacter baumannii* was defined in accordance with Sepsis 3 definitions [59]. Cefiderocol was administered at standard doses, that is, 2 g every 8 h with a 3 h infusion, to provide >90% probability of target attainment (PTA) for Gram-negative bacteria with Cefiderocol MIC values up to 4 μg/mL [10,21]. In contrast, a dose of 2 g every 6 h was chosen for patients with augmented renal clearance (ARC, CrCl > 120 mL/min) in the absence of a previous determination of the sensitivity of the individual CRAB strain(s) isolated from each patient, unavailable at our site until recently [22].

None of the included patients were in their pediatric age, pregnant, or with previously known immune suppression such as chronic steroid therapy, other immunosuppressive therapies for solid organ transplant, or autoimmune diseases, cancer chemotherapy, allogeneic hematopoietic stem cell transplantation, or supervening infection with SARS-CoV-2.

Continuous variables are presented as medians with IQRs, categorical variables are shown as frequencies with percentages, and groups were compared using the Mann–Whitney test and the Fisher’s exact test, respectively. Univariate and multivariable logistic regression analyses were performed to evaluate the prognostic value of age, treatment (monotherapy vs. combination), SAPS II, SOFA, lymphocytopenia, reactivation, CD4, the ratio CD8/CD19, and cholinesterase for mortality. In the multivariable models of logistic regression, we used a backward procedure with a threshold of *p* = 0.20. This procedure allows for the exclusion of variables that are not associated with the outcome measure at a given significance level. The discriminant capability of the final model was evaluated by the area under the curve (AUC) of the receiver operating characteristic (ROC) curve and the Hosmer and Lemeshow test for the goodness of fit.

## Figures and Tables

**Table 1:** Selected features of patients in the survivor and death groups.

	Survival (*n* = 20)		Death (*n* = 16)		Overall (*n* = 36)		*p* Value *
**Age, years**	52	40.5–68.5	73	64.5; 78	64.5	46.5–75.5	**0.003**
Sex (n. %)							
Male	15	75	11	69	26	72	
Female	5	25	5	31	10	28	0.677
Length of stay, days	31	20–47	26	12; 35	30	18–38	0.149
Diagnosis (n. %)							
Septic shock	3	15	7	43.8	10	27.8	
Polytrauma	7	35	2	12.5	9	25	
Subarachnoid hemorrhage/ thromboembolism	8	40	1	6.3	9	25	
Hemorrhagic shock	1	5	4	25	5	13.9	
Respiratory failure	1	5	2	12.5	3	8.3	0.02
Sepsis (n. %)	17	85	16	100	33	91.7	0.106
Treatment (n. %)							
Monotherapy (n. %)	10	50	9	56.3	19	52.8	
Combination (n. %)	10	50	7	43.7	17	47.2	0.709
SAPS II, median (IQR)	38.5	28–46.5	51	35–64	42	32–55	0.048
SOFA, median (IQR)	5.5	4–7.5	8	6–11.5	6	4–9	0.018
Lymphocytopenia (n. %)							
Absent	13	68.4	1	6.3	14	40	
Temporary	6	31.6	2	12.5	8	22.9	
Persistent	0	0	13	81.2	13	37.1	<0.01
CD4-T, <400 c/µL	10	62.5	12	80	22	71	0.283
CD8/CD19	3	18.8	3	20	6	19.4	0.93
Cholinesterase, <1600 U/L	3	15	14	87.5	17	47.2	<0.01
Reactivation (n. %)	2	14.3	13	61.9	15	42.7	0.005

Data are presented as median and IQR or number and percentage. * Fisher exact test or Mann–Whitney, as appropriate.

**Table 2:** Final model of logistic regression for independent predictors of death.

	Non-Adjusted		Adjusted *	
	OR (95% CI)	*p* Value	OR (95% CI)	*p* Value
Age (per 5-year increase)	1.50 (1.11–2.02)	0.008	1.42 (0.98–2.05)	0.063
Sex: female vs. male	1.36 (0.32–5.89)	0.678		
Treatment: monotherapy vs. combination	1.29 (0.34–4.82)	0.709		
SAPS II (per 1 point increase)	1.05 (1.01–1.10)	0.042		
SOFA (per 1 point increase)	1.37 (1.06–1.76)	0.017		
Lymphocytopenia (persistent or temporary vs. absent)	32.5 (3.45–306.35)	0.002	18.16 (0.87–381.38)	0.062
Reactivation	9.75 (1.72–55.37)	0.01		
CD4 < 400 c/µL	2.4 (0.47–12.13	0.29		
CD8/CD19 ratio > 2.2	1.08 (0.18–6.44)	0.93		
Cholinesterase < 1600 U/L	9.67 (5.79–271.63)	<0.01	9.77 (0.77–123.70)	0.078

* stepwise with *p* = 0.02.

## Data Availability

The data that support the findings of this study are available on request from the corresponding author.
